# Comparison of the end-of-life decisions of patients with hospital-acquired pneumonia after the enforcement of the life-sustaining treatment decision act in Korea

**DOI:** 10.1186/s12910-023-00931-y

**Published:** 2023-07-18

**Authors:** Ae-Rin Baek, Sang-Bum Hong, Soohyun Bae, Hye Kyeong Park, Changhwan Kim, Hyun-Kyung Lee, Woo Hyun Cho, Jin Hyoung Kim, Youjin Chang, Heung Bum Lee, Hyun-Il Gil, Beomsu Shin, Kwang Ha Yoo, Jae Young Moon, Jee Youn Oh, Kyung Hoon Min, Kyeongman Jeon, Moon Seong Baek, and the Korean HAP/VAP Study Group

**Affiliations:** 1 Division of Allergy and Pulmonary Medicine, Department of Internal Medicine, Soonchunhyang University Bucheon Hospital, Bucheon, Korea; 2 Department of Pulmonary and Critical Care Medicine, Asan Medical Center, University of Ulsan College of Medicine, Seoul, Korea; 3 Department of Integrated Internal Medicine, Myongji Hospital, Hanyang University College of Medicine, Goyang, Korea; 4 Division of Pulmonary and Critical Care Medicine, Department of Internal Medicine, Ilsan Paik Hospital, Inje University College of Medicine, Ilsan, Korea; 5 Department of Internal Medicine, Jeju National University Hospital, Jeju National University School of Medicine, Jeju, Korea; 6 Department of Internal Medicine, Division of Pulmonology, Allergy and Critical Care Medicine, Busan Paik Hospital, Inje University College of Medicine, Busan, Korea; 7 Division of Allergy, Pulmonary and Critical Care Medicine, Department of Internal Medicine, Pusan National University Yangsan Hospital, Pusan National University School of Medicine, Busan, Korea; 8 Division of Respiratory and Critical Care Medicine, Department of Internal Medicine, Ulsan University Hospital, University of Ulsan College of Medicine, Ulsan, Korea; 9 Division of Pulmonary and Critical Care Medicine, Department of Internal Medicine, Inje University Sanggye Paik Hospital, Seoul, Korea; 10 Department of Internal Medicine, Research Center for Pulmonary Disorders, Jeonbuk National University Medical School, Jeonju, Korea; 11 Division of Pulmonary and Critical Care Medicine, Department of Internal Medicine, Kangbuk Samsung Hospital, Sungkyunkwan University School of Medicine, Seoul, Korea; 12 Division of Pulmonary and Critical Care Medicine, Department of Medicine, Samsung Changwon Hospital, Sungkyunkwan University School of Medicine, Changwon, Korea; 13 Division of Pulmonary, Allergy, and Critical Care Medicine, Department of Internal Medicine, Konkuk University School of Medicine, Seoul, Korea; 14 Division of Pulmonary, Allergy, and Critical Care Medicine, Department of Internal Medicine, Chungnam National University College of Medicine, Sejong Hospital, Sejong, Korea; 15 Division of Pulmonary, Allergy, and Critical Care Medicine, Department of Internal Medicine, Korea University Guro Hospital, Seoul, Korea; 16 Division of Pulmonary and Critical Care Medicine, Department of Medicine, Samsung Medical Center, Sungkyunkwan University School of Medicine, Seoul, Korea; 17 Division of Pulmonary and Critical Care Medicine, Department of Internal Medicine, Chung-Ang University Hospital, Chung-Ang University College of Medicine, 102, Heukseok-Ro, Dongjak-Gu, Seoul, 06973 Republic of Korea

**Keywords:** Terminal care, Resuscitation orders, Withholding treatment, Healthcare-associated pneumonia

## Abstract

**Background:**

Although the Life-Sustaining Treatment (LST) Decision Act was enforced in 2018 in Korea, data on whether it is well established in actual clinical settings are limited. Hospital-acquired pneumonia (HAP) is a common nosocomial infection with high mortality. However, there are limited data on the end-of-life (EOL) decision of patients with HAP. Therefore, we aimed to examine clinical characteristics and outcomes according to the EOL decision for patients with HAP.

**Methods:**

This multicenter study enrolled patients with HAP at 16 referral hospitals retrospectively from January to December 2019. EOL decisions included do-not-resuscitate (DNR), withholding of LST, and withdrawal of LST. Descriptive and Kaplan–Meier curve analyses for survival were performed.

**Results:**

Of 1,131 patients with HAP, 283 deceased patients with EOL decisions (105 cases of DNR, 108 cases of withholding of LST, and 70 cases of withdrawal of LST) were analyzed. The median age was 74 (IQR 63–81) years. The prevalence of solid malignant tumors was high (32.4% vs. 46.3% vs. 54.3%, *P* = 0.011), and the ICU admission rate was lower (42.9% vs. 35.2% vs. 24.3%, *P* = 0.042) in the withdrawal group. The prevalence of multidrug-resistant pathogens, impaired consciousness, and cough was significantly lower in the withdrawal group. Kaplan–Meier curve analysis revealed that 30-day and 60-day survival rates were higher in the withdrawal group than in the DNR and withholding groups (log-rank *P* = 0.021 and 0.018). The survival of the withdrawal group was markedly decreased after 40 days; thus, the withdrawal decision was made around this time. Among patients aged below 80 years, the rates of EOL decisions were not different (*P* = 0.430); however, mong patients aged over 80 years, the rate of withdrawal was significantly lower than that of DNR and withholding (*P* = 0.001).

**Conclusions:**

After the LST Decision Act was enforced in Korea, a DNR order was still common in EOL decisions. Baseline characteristics and outcomes were similar between the DNR and withholding groups; however, differences were observed in the withdrawal group. Withdrawal decisions seemed to be made at the late stage of dying. Therefore, advance care planning for patients with HAP is needed.

**Supplementary Information:**

The online version contains supplementary material available at 10.1186/s12910-023-00931-y.

## Background

The definition of well-dying can be highly subjective; however, it is often referred to as death with dignity. This notion includes concepts such as peacefulness, physical comfort, autonomy, preparedness, connectedness with loved ones, and awareness [1]. The Korean guidelines for comfort care and withdrawing/withholding treatment in the intensive care unit (ICU) also emphasize sufficient family interview, communication, individualization, symptom control, family participation, and bereavement care as key factors [2]. Although people with imminent death prefer death with dignity, the number of patients dying in the ICU is increasing [3]. They often receive futile ICU care, and the costs are substantial [4].

Recently, interest in well-dying has been increasing in Korea, which may be attributed to the introduction of legislation related to hospice and palliative care and decisions on life-sustaining treatment (LST) for patients at the end-of-life (EOL) [5]. Before the relevant law was introduced, EOL decisions were made based on the physician’s judgment and the family members’ request or agreement [6]. Previously, when family members or patients requested discharge against a physician’s decision, this was regarded as “discharge against medical advice” and was often accepted. However, after the Boramae Hospital Incident in 1997, in which physicians who discharged a mechanically ventilated patient with brain hemorrhage at the request of the patient’s wife were sentenced for aiding a murder, physicians now make EOL decisions conservatively. However, in the so-called Grandmother Kim case in 2008, the physician refused the request for withdrawal of LST from the patient’s family, and the Supreme Court finally ordered the withdrawal [7]. Following these prominent cases involving LST, for death with dignity and self-determination, the LST Decision Act was enforced in 2018.

Since the enforcement of the law, several changes such as improved quality of death [8] and increased rate of self-determination [9] were reported. However, the law did not reduce the incidence of cardiopulmonary resuscitation (CPR) [10], and EOL decisions were made during the dying process [11]. The clinical course according to the EOL decision remains uncertain because most studies did not differentiate the withdrawal and withholding of LST.

Pneumonia is one of the main causes of death in the hospital. Previous studies demonstrated that hospital-acquired pneumonia (HAP) accounted for around 7% of hospital deaths [12], and the attributable mortality of ventilator-associated pneumonia (VAP) was 13% [13]. The dying process associated with pneumonia results in a situation where patients and their family may struggle with making an unexpected EOL decision. The EOL decision for patients with pneumonia is often difficult because of the uncertainty about the patients' wishes and their prognoses [14]. As there are limited data on the EOL decision of patients with HAP, we aimed to examine clinical characteristics and course according to the EOL decision of patients with HAP.

## Methods

### Study design and patients

This was a multicenter cohort study conducted at 16 referral hospitals from January 1 to December 31, 2019 in Korea. The data of patients aged over 19 years were retrospectively screened using electronic medical records. We enrolled patients with more than 3 consecutive days of hospitalization and pneumonia-related diagnostic codes (International Classification of Diseases-10 code, J13–J18, J85). Among them, we included patients with the following criteria according to the international guidelines for HAP [15]: presence of lung infiltrates (new or progressive) and clinical signs of pneumonia (new onset of fever, purulent sputum, leukocytosis, and a decline in oxygenation). Among patients with HAP, we excluded those without EOL decisions or those who were discharged alive.

This study was approved by the Institutional Review Board and Ethics Committee of Soonchunhyang University Bucheon Hospital (No. 2020–03-037–003) and the local committees of all other participating centers. The need for informed consent was waived by the institutional review boards of Soonchunhyang University Bucheon Hospital, Asan Medical Center, Myongji Hospital, Inje University Ilsan Paik Hospital, Jeju National University Hospital, Inje University Busan Paik Hospital, Pusan National University Yangsan Hospital, Ulsan University Hospital, Inje University Sanggye Paik Hospital, Jeonbuk National University Hospital, Kangbuk Samsung Hospital, Samsung Changwon Hospital, Konkuk University Medical Center, Chungnam National University Sejong Hospital, Korea University Guro Hospital, and Samsung Medical Center due to the retrospective nature of the study. All methods were carried out in accordance with the Helsinki Declaration and the relevant guidelines and regulations.

### Data collection

The following data at HAP diagnosis were collected by trained coordinators at each center: age, sex, body mass index, comorbidities, reasons for admission (diagnostic work-up, medical disease treatment, elective operation, and emergency operation), Charlson Comorbidity Index (CCI), Clinical Frailty Scale (CFS), Sequential Organ Dysfunction Assessment (SOFA) score, location of HAP diagnosis, artificial airway, tube feeding, impaired consciousness, impaired cough, sepsis, VAP, multidrug-resistant (MDR) pathogens, ICU admission, in-hospital mortality, and discharge destination (home or transfer to step-down facility).

For ICU-admitted patients, the following data were collected: reasons for ICU admission (respiratory failure or septic shock), SOFA scores within 24 h and at 48 h, therapy within 24 h, therapy during ICU stay, ICU duration, mechanical ventilation (MV) duration, and ICU mortality. Furthermore, events during ICU stay, including VAP, catheter-related infection, urinary tract infection, acute respiratory distress syndrome (ARDS), arrhythmia, bleeding requiring intervention, and CPR, were recorded.

### Definitions

The LST Decision Act defines several terms [5]. The EOL process indicates a state of imminent death, in which there is no possibility of revitalization or recovery despite treatment, and symptoms worsen rapidly. Patients at the EOL were identified by the doctor in charge and a medical specialist in the relevant field. LST is defined as medical treatment including CPR, hemodialysis, anticancer drugs, and MV for patients at the EOL. The Physician Order for Life-Sustaining Treatment (POLST), including the withholding and withdrawal of LST, is prepared according to the intention of patients. For patients with a do-not-resuscitate (DNR) order, only CPR was not performed in the case of cardiac arrest. EOL decisions including the DNR order and POLST were made at any time during admission when the patients were considered at the EOL.

VAP is defined as pneumonia occurring at least 48 h after endotracheal intubation [15]. We considered VAP as pneumonia affecting patients with artificial airway in the ICU. According to the Sepsis-3 definition, we considered sepsis as an acute change in the total SOFA score of more than 2 points for patients with HAP [16]. Septic shock was considered when there was a persistent requirement of vasopressors for sepsis patients with an elevated serum lactate level (> 2 mmol/L).

### Statistical analysis

Categorical variables are presented as the number (percentage), and continuous variables are presented as the median (interquartile range [IQR]) or mean (standard deviation). Data on categorical variables were compared using Chi-square test or Fisher’s exact test. To compare continuous variables, one-way analysis of variance (ANOVA) or Kruskal–Wallis test was used as appropriate. Pairwise comparison for each EOL decision was performed using the Bonferroni method. In addition, Kaplan–Meier curve analysis for 30-day and 60-day survival was performed. The Kaplan–Meier curves between three groups were compared by log-rank test. Time from HAP diagnosis to death was analyzed by linear regression, and adjusted variables were age, sex, BMI, chronic liver disease, solid malignant tumors, CCI, CFS, SOFA score, artificial airway, tube feeding, impaired consciousness, impaired cough, VAP, and MDR pathogens. We performed all statistical analyses using R version 4.2.2 (R Foundation for Statistical Computing), and *P* values less than 0.05 were considered statistically significant.

## Results

### Patient characteristics

There were 1,131 patients with HAP, and 780 patients without EOL decisions were excluded (Supplementary Fig. 1). Among 351 patients, 68 patients who were discharged to their home or a step-down facility were excluded. We analyzed 283 deceased patients with a POLST (108 cases of withholding and 70 cases of withdrawal of LST) or DNR order (n = 105). The overall patients’ median age was 74 (IQR 63–81) years, and 65.7% of them were male. The CCI and CFS were 6 (IQR 4–8) and 6 (IQR 4–7), respectively. Sepsis occurred in 70.3% of patients, and 35% of them were fed via a tube. Table 1 shows a comparison of the characteristics of three groups according to the EOL decision, and the results of pairwise comparison are shown in Supplementary Table 1. The prevalence of solid malignant tumors was the highest in the withdrawal group (32.4% vs. 46.3% vs. 54.3%, *P* = 0.011), and it was significantly higher in the withdrawal group than in the DNR group (*P* = 0.019). The CCI was lower in the DNR group than in the withdrawal group (5 [IQR 4–7] vs. 5 [IQR 5–8], *P* = 0.031). The prevalence of MDR pathogens, impaired consciousness, and cough was significantly lower in the withdrawal group. There was no significant difference in age, sex, reasons for admission, CFS, SOFA score at HAP diagnosis, tube feeding, and sepsis. The rate of ICU admission was significantly lower in the withdrawal group (42.9% vs. 35.2% vs. 24.3%, *P* = 0.042). There was no difference in hospital length of stay; however, time from onset of HAP to death was longer in the withdrawal group than in the other groups (10 [IQR 5–25] vs. 14 [IQR 6–26] vs. 18.5 [IQR 7–43], *P* = 0.036). In addition, 30-day mortality was lower in the withdrawal group (81.0% vs. 78.7% vs. 62.9%, *P* = 0.015). However, 60-day mortality did not show a statistically significant difference between the three groups (95% vs. 89.8% vs. 85.7%, *P* = 0.092).

**Table 1:** Baseline characteristics of deceased patients according to the EOL decision

Variables	DNR (*n* = 105)	Withholding (*n* = 108)	Withdrawal (*n* = 70)	*P*
Age (years)	73 (62–81)	75 (64–82.5)	70.5 (59–78)	0.172
Male, n (%)	69 (65.7)	70 (64.8)	48 (68.6)	0.871
Body mass index (kg/m^2^)	20.9 (18.5–24.5)	21.9 (19.6–24.2)	21.9 (18.9–23.8)	0.728
Comorbidities, n (%)				
Diabetes	24 (22.9)	36 (33.3)	21 (30.0)	0.229
Cardiovascular disease	31 (29.5)	19 (17.6)	17 (24.3)	0.122
Chronic lung disease	19 (18.1)	10 (9.3)	15 (21.4)	0.060
Chronic neurological disease	26 (24.8)	38 (35.2)	16 (22.9)	0.123
Chronic kidney disease	23 (21.9)	18 (16.7)	8 (11.4)	0.195
Chronic liver disease	10 (9.5)	9 (8.3)	14 (20.0)	0.042
Hematological malignancy	17 (16.2)	14 (13.0)	15 (21.4)	0.327
Solid malignant tumors	34 (32.4)	50 (46.3)	38 (54.3)	0.011
Connective tissue disease	3 (2.9)	5 (4.6)	1 (1.4)	0.530
Immunocompromized	11 (10.5)	12 (11.1)	3 (4.3)	0.259
Reasons for admission, n (%)				0.059
Diagnostic work-up	16 (15.2)	9 (8.3)	6 (8.6)	
Medical disease treatment	69 (65.7)	80 (74.1)	52 (74.3)	
Elective operation	8 (7.6)	9 (8.3)	11 (15.7)	
Emergency operation	12 (11.4)	10 (9.3)	1 (1.4)	
Charlson Comorbidity Index	5 (4–7)	6 (5–8)	6 (5–8)	0.023
Clinical Frailty Scale	6 (4–7)	6 (4–8)	6 (4–7)	0.562
SOFA score	6 (4–10)	5 (3–9)	6 (4–9)	0.515
Location of diagnosis, n (%)				0.386
General ward	74 (70.5)	85 (78.7)	52 (74.3)	
ICU	31 (29.5)	23 (21.3)	18 (25.7)	
Artificial airway, n (%)	31 (29.5)	21 (19.4)	14 (20.0)	0.165
Tube feeding, n (%)	40 (38.1)	38 (35.2)	21 (30.0)	0.545
Impaired consciousness, n (%)	52 (49.5)	41 (38.0)	21 (30.0)	0.030
Impaired cough, n (%)	40 (38.1)	41 (38.0)	11 (15.7)	0.003
Sepsis, n (%)	78 (74.3)	78 (72.2)	43 (61.4)	0.163
Ventilator-associated pneumonia, n (%)	26 (24.8)	16 (14.8)	12 (17.1)	0.162
MDR pathogens, n (%)	47 (44.8)	34 (31.5)	15 (21.4)	0.005
ICU admission, n (%)	45 (42.9)	38 (35.2)	17 (24.3)	0.042
Hospital length of stay, days	29 (18–51)	33 (21–51)	33.5 (21–59)	0.267
Onset of HAP to death, days	10 (5–25)	14 (6–26)	18.5 (7–43)	0.036
30-day mortality, n (%)	85 (81.0)	85 (78.7)	44 (62.9)	0.015
60-day mortality, n (%)	100 (95.2)	97 (89.8)	60 (85.7)	0.092

Values are presented as the median (IQR) or number (%)

*EOL* End-of-life, *DNR* Do not resuscitate, *SOFA* Sequential Organ Dysfunction Assessment, *MDR* Multidrug-resistant, *ICU* Intensive care unit, *HAP* Hospital-acquired pneumonia

### ICU patient characteristics

Among 100 patients admitted to the ICU, there were 45 patients with a DNR, 38 patients with withholding of LST, and 17 patients with withdrawal of LST. The main reasons for ICU admission were respiratory failure (57%) and septic shock (36%) (Table 2). During ICU stay, there was no significant difference in high-flow nasal cannula (HFNC), MV, and extracorporeal membrane oxygenation (ECMO); however, renal replacement therapy (RRT) was more common in the DNR group (46.7%) than in the withholding (15.8%) and withdrawal (29.4%) groups (*P* = 0.011). Pairwise comparison demonstrated that RRT was significantly more prevalent in the DNR group than in the withholding group (*P* = 0.018), and there was no difference between the DNR and withdrawal groups (*P* = 1.000) (Supplementary Table 2). The incidence rates of VAP, catheter-related infection, urinary tract infection, and bleeding requiring intervention were not significantly different between the groups. Although EOL decisions were made, 11.1%, 7.9%, and 5.9% of patients in the DNR, withholding, and withdrawal groups received CPR, respectively. There were no differences in the ICU and MV duration. ICU mortality was significantly higher in the DNR group than in the other groups (88.9% vs. 71.1% vs. 64.7%, *P* = 0.048).

**Table 2:** Characteristics of ICU patients

Variables	DNR (*n* = 45)	Withholding (*n* = 38)	Withdrawal (*n* = 17)	*P*
Reasons for ICU admission, n (%)				0.503
Respiratory failure	22 (48.9)	24 (63.2)	11 (64.7)	
Septic shock	18 (40.0)	13 (34.2)	5 (29.4)	
Others	5 (11.1)	1 (2.6)	1 (5.9)	
SOFA score				
Within 24 h	8.9 ± 4.1	8.5 ± 3.6	9.3 ± 3.7	0.770
At 48 h	10.9 ± 4.4	9.8 ± 4.8	9.8 ± 3.9	0.521
Therapy within 24 h, n (%)				
Vasopressors	36 (80.0)	24 (63.2)	11 (64.7)	0.194
Inotropes	2 (4.4)	0 (0.0)	2 (11.8)	0.093
HFNC	17 (37.8)	15 (39.5)	5 (29.4)	0.767
MV	29 (64.4)	21 (55.3)	10 (58.8)	0.692
Therapy during ICU stay, n (%)				
HFNC	19 (42.2)	20 (52.6)	6 (35.3)	0.431
MV	35 (77.8)	26 (68.4)	13 (76.5)	0.655
RRT	21 (46.7)	6 (15.8)	5 (29.4)	0.011
ECMO	1 (2.2)	0 (0.0)	2 (11.8)	0.074
Events during ICU stay, n (%)				
Ventilator-associated pneumonia	8 (17.8)	4 (10.5)	3 (17.6)	0.698
Catheter-related infection	6 (13.3)	1 (2.6)	0 (0.0)	0.098
Urinary tract infection	4 (8.9)	3 (7.9)	2 (11.8)	0.810
ARDS	9 (20.0)	13 (34.2)	3 (17.6)	0.294
Arrhythmia	14 (31.1)	5 (13.2)	2 (11.8)	0.099
Bleeding requiring intervention	6 (13.3)	2 (5.3)	0 (0.0)	0.225
Cardiopulmonary resuscitation	5 (11.1)	3 (7.9)	1 (5.9)	0.902
ICU duration	8 (5–20)	9.5 (5–17)	15 (6–32)	0.399
MV duration	5 (1–15)	5 (0–12)	4 (3–15)	0.879
ICU mortality	40 (88.9)	27 (71.1)	11 (64.7)	0.048
30-day mortality after ICU admission	38 (84.4)	32 (84.2)	9 (52.9)	0.026
60-day mortality after ICU admission	40 (88.9)	36 (94.7)	15 (88.2)	0.581

Values are presented as the mean ± SD, median (IQR), or number (%)

*ICU* Intensive care unit, *DNR* Do not resuscitate, *SOFA* Sequential Organ Dysfunction Assessment, *HFNC* High-flow nasal cannula, *MV* Mechanical ventilation, *RRT* Renal replacement therapy, *ECMO* Extracorporeal membrane oxygenation, *ARDS* Acute respiratory distress syndrome

### Linear regression analysis and Cox proportional hazards regression models

Multivariable linear regression analysis showed that time from onset of HAP to death was 13 days shorter in the DNR group than in the withdrawal group (-13.2 days, 95% CI -24.9 to -1.6, *P* = 0.026) (Table 3). In addition, it was 5 days shorter in the withholding group than in the withdrawal group; however, there was no statistical significance (*P* = 0.365).

**Table 3:** Univariable and multivariable linear regression analysis for time from onset of HAP to death

Variable	Univariable		Multivariable^a^	
	Estimate (95% CI)	*P*	Estimate (95% CI)	*P*
EOL decisions				
DNR	-11.9 (-23.0 to -0.8)	0.035	-13.2 (-24.9 to -1.6)	0.026
Withholding	-4.1 (-15.1 to 7.0)	0.470	-5.2 (-16.5 to 6.1)	0.365
Withdrawal	1 (Reference)		1 (Reference)	

*HAP* Hospital-acquired pneumonia, *CI* Confidence interval, *DNR* Do not resuscitate

^a^Adjusted for age, sex, BMI, chronic liver disease, solid malignant tumors, Charlson Comorbidity Index, Clinical Frailty Scale, SOFA score, artificial airway, tube feeding, impaired consciousness, impaired cough, ventilator-associated pneumonia, and MDR pathogens

### Kaplan–Meier curve analysis

**Fig. 1:**
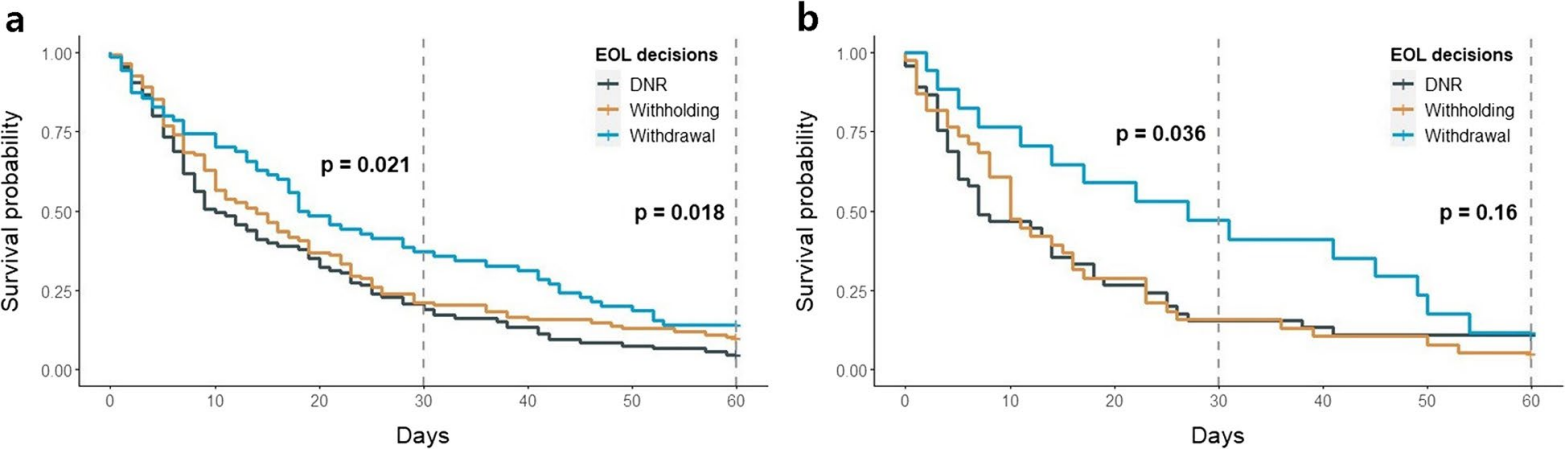
Kaplan–Meier curve analysis for 60-day survival. **A** Overall patients. **B** ICU patients

The results of Kaplan–Meier curve analysis for 30-day and 60-day survival are shown in Fig. 1. Among overall patients, 30-day and 60-day survival rates were significantly higher in the withdrawal group than in the DNR and withholding groups (log-rank *P* = 0.021 and 0.018). Among ICU patients, 30-day survival was significantly higher in the withdrawal group than in the withholding and DNR groups (log-rank *P* = 0.036). However, 60-day mortality was not significantly different between the three groups (log-rank *P* = 0.160). Kaplan–Meier curve analysis demonstrated that the survival of the withdrawal group was markedly decreased after 40 days (Fig. 1 A and B).

Among patients with low CCI (1–5), 60-day survival was significantly higher in the withdrawal group (log-rank *P* = 0.038); however, among those with high CCI (≥ 6), there was no significant difference between the three groups (log-rank *P* = 0.260). Subgroup analysis of patients with any malignancy (hematologic malignancy and solid tumors) compared with those without malignancy showed different patterns of Kaplan–Meier curves, which demonstrated a substantial decrease in survival after 40 days (Supplementary Fig. 2).

### Subgroup analysis of EOL decisions

**Fig. 2:**
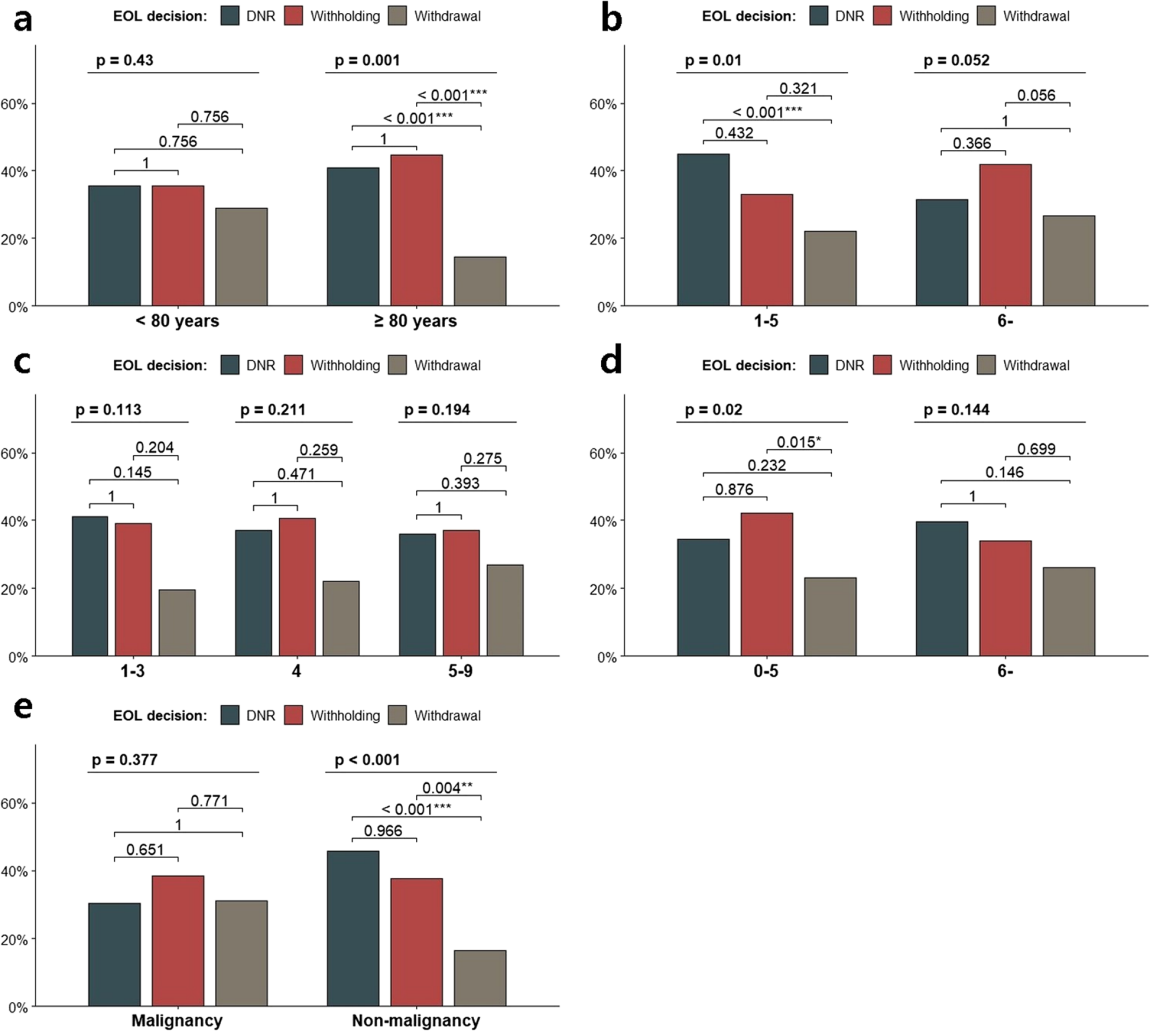
Rate of EOL decisions. EOL: end-of-life. **A** According to age (Chi-square for independence, *P* = 0.034). **B** According to Charlson Comorbidity Index (CCI) (Chi-square for independence, *P* = 0.070). **C** According to Clinical Frailty Scale (CFS) (Chi-square for independence, *P* = 0.828). **D** According to Sequential Organ Dysfunction Assessment (SOFA) score (Chi-square for independence, *P* = 0.363). **E** According to malignancy (Chi-square for independence, *P* = 0.005)

The results of subgroup analysis for EOL decisions according to age, CCI, CFS, SOFA score, and malignancy are shown in Fig. 2. The EOL decisions of patients with HAP were different depending on their age (*P* = 0.034). Among patients aged below 80 years, the rates of the EOL decisions were not significantly different (*P* = 0.430); however, among patients aged over 80 years, the rate of withdrawal was significantly lower than that of DNR and withholding (*P* = 0.001). In terms of comorbidities, patients with low CCI had a lower rate of withdrawal compared with DNR (*P* = 0.010). There was no significant difference among patients with high CCI. In addition, there were no differences in EOL decisions according to CFS and SOFA score (*P* = 0.828 and 0.363, respectively). However, among patients with low SOFA score, the rate of withdrawal was significantly lower than that of withholding (*P* = 0.015). In terms of malignancy (hematologic malignancy and solid tumors), there were no differences in EOL decisions (*P* = 0.377); however, the rate of withdrawal was significantly low among patients without malignancy (*P* < 0.001).

## Discussion

In this multicenter study, we evaluated the characteristics of patients with HAP according to the EOL decision, including the factors associated with the decision. The withdrawal group was different in terms of solid malignant tumors, impaired consciousness, and cough. Kaplan–Meier curve analysis revealed that the DNR and withholding groups were similar; however, the withdrawal group showed a significantly higher survival rate. Notably, Kaplan–Meier curve analysis demonstrated that the survival of the withdrawal group was markedly decreased after 40 days. The EOL decisions of patients with HAP were different depending on their age and comorbid malignancy; the rate of withdrawal was significantly lower among patients aged over 80 years and those without malignancy.

Although a year has passed since the LST Decision Act was enforced, we found that the DNR order was still common. A DNR order is much easier to authorize than a POLST, which may explain why it is more common. In cases where a patient with a specific medical condition is unable to express his/her intention, if two or more family members can provide an identical statement indicating that the patient had previously expressed an intention to discontinue LST, it is considered that the patient has made an EOL decision [5]. If it is impossible to infer the patient’s intention, the EOL decision must be confirmed by consensus of all legal family members. Furthermore, to implement a POLST, the establishment of an institutional ethics committee is essential. Such an institution or a specific legal process is not required for a DNR order. However, a DNR decision is likely made following a discussion between the physician and family members irrespective of the patient’s will [17]. Furthermore, a DNR order is a document not covered by the LST Decision Act. Physicians should consider the patient’s preference in terms of self-determination, and the law needs to be improved to reduce the hurdles of the POLST.

For patients with a DNR order, LST was not restricted except for CPR. However, there were no significant differences in baseline characteristics, hospital length of stay, mortality, ICU admission rate, and therapy during ICU stay between the DNR and withholding groups. Notably, the Kaplan–Meier curves of the DNR and withholding groups were similar. We observed that there were differences only in CCI and RRT; compared with the withholding group, the DNR group had a lower CCI and underwent RRT more frequently. Therefore, the withholding and DNR groups received other medical treatments such as vasopressors and MV without limitations in the course of death. The purpose of the relevant law is to preserve dignity by ensuring the best interests of patients and respecting their self-determination [5]. It is necessary to review whether a POLST merely reflects a conventional DNR decision.

Withdrawal of LST is a more active action than withholding of LST because it is executed when the patient or caregiver is prepared to accept imminent death. Although it is known that withdrawal and withholding are not ethically different [18], Asian physicians perceive withholding and withdrawal differently, and they seldom make withdrawal decisions [19, 20]. In agreement with previous studies, our study showed that the incidence of withdrawal of LST was low, and the Kaplan–Meier curves demonstrated significantly different patterns between the withdrawal group and the withholding group. Interestingly, the survival of the withdrawal group remained relatively higher until around day 40 of HAP onset before rapidly decreasing. Therefore, the withdrawal decision was made in the later stage rather than the early stage of patients with HAP. Furthermore, compared with the withholding group, the withdrawal group had a higher prevalence of solid malignant tumors and was more likely to receive RRT. Therefore, the majority of patients with malignant disease died after withdrawing LST at a later stage while receiving active treatment. Although some studies have reported that the LST Decision Act is associated with an early EOL decision [8, 9], the withdrawal of LST still appears to be delayed.

There were several differences between the withdrawal group and other groups. Based on the findings, more cancer patients were included in the withdrawal group. According to Lee et al., a higher rate of self-determination in EOL decisions was observed among cancer patients compared with non-cancer patients [21]. A possible explanation is that judgment of the disease trajectory of cancer is easier compared with that of other benign diseases [22]. On the other hand, among patients without any malignancy, the rate of withdrawal was significantly low. Another notable finding was that withdrawal of LST was decreased among those older than 80 years. Park et al. found that the odds ratio of self-determination in LST was significantly reduced for patients over 80 years of age compared with those under 80 years of age [11]. Furthermore, family members still play a major role in EOL decisions in Korea; a nationwide study showed that family determination influenced the EOL decisions of 90% of non-cancer patients [21]. Family members would experience grief due to the resulting death after a withdrawal decision [23]. Therefore, the reason for the decrease in the withdrawal of LST among older patients may be attributed to the family’s desire to avoid withdrawal.

To avoid unnecessary suffering from futile LST, advance directives are crucial. As of 3 years after the LST Decision Act was enacted, around 1.5 million people (3.5% of adults) in Korea have prepared an advance directive [24]. For death with dignity, in-depth communication between the patient, family members, and physician through advance directives is more important than documentation only [6]. In Korea, EOL decisions are usually made when death is imminent [11, 25], as it is difficult for doctors to accurately predict the remaining survival period of terminally ill patients. Moreover, the LST Decision Act does not apply to patients with a persistent vegetative state, which is contrary to the situation in Japan or Taiwan [26]. Therefore, experts emphasize the active role of the hospital ethics committee, which can help resolve ethical issues during the process of making EOL decisions [27]. For well-dying, institutional improvements to remove various obstacles to law enforcement along with the preparation of advance directives and monitoring of the performance of hospital ethics committees must be collectively implemented.

There are several limitations in our study. First, due to the retrospective nature of the study, we could not collect detailed data associated with the EOL decision (e.g., timing, LST items, and parties involved). Although we did not report the timing or subjects of EOL decisions, similar to the findings of studies conducted after the Act was enforced [11, 21, 25], EOL decisions would have been mostly made by family members during the dying process. Second, we did not examine economic status or religion, which may affect EOL decisions [19]. Furthermore, it may be difficult to generalize our findings to all hospitalized patients because this study enrolled patients diagnosed with HAP. However, HAP is the leading cause of death among patients with nosocomial infection [28], particularly older patients with comorbidities [29]. Therefore, this study is helpful for understanding the dying process of hospitalized patients. Furthermore, the results of our study suggest that the LST Decision Act should be further improved.

## Conclusions

After the LST Decision Act was implemented in Korea, a DNR order was still common in EOL decisions. Baseline characteristics and outcomes were similar between the DNR and withholding groups; however, differences were observed in the withdrawal group. The withdrawal group included a large proportion of patients with solid malignant tumors. The withdrawal decision was less common among older patients over 80 years of age, and it seemed to be made at the late stage of the dying process. Therefore, to avoid futile LST, advance care planning is needed.

## Supplementary Information

**Additional file 1: Fig. S1.** Flowchart. HAP: hospital-acquired pneumonia, EOL: end-of-life, DNR: do not resuscitate. **Fig. S2.** Kaplan-Meier curve analysis for 60-day survival according to malignancy (a) Patients with malignancy. (b) Patients without malignancy.

**Additional file 2: Supplementary Table 1.** Pairwise comparison of the overall cohort. **Supplementary Table 2.** Pairwise comparison of ICU patients.

## Data Availability

The datasets used and analyzed in the current study are available from the corresponding author upon reasonable request.

## Abbreviations

LST: Life-sustaining treatment
HAP: Hospital-acquired pneumonia
EOL: End-of-life
CPR: Cardiopulmonary resuscitation
DNR: Do-not-resuscitate
MDR: Multidrug-resistant
ICU: Intensive care unit
CCI: Charlson Comorbidity Index
CFS: Clinical Frailty Scale
SOFA: Sequential Organ Dysfunction Assessment
VAP: Ventilator-associated pneumonia
MV: Mechanical ventilation
ARDS: Acute respiratory distress syndrome
POLST: Physician Order for Life-Sustaining Treatment
IQR: Interquartile range
ANOVA: One-way analysis of variance
