# Supplementary material for: Comparison of the end-of-life decisions of patients with hospital-acquired pneumonia after the enforcement of the life-sustaining treatment decision act in Korea
Source: BMC Med Ethics. 2023 Jul 18;24:52. doi: 10.1186/s12910-023-00931-y (PMC10353089; doi:10.1186/s12910-023-00931-y)

**Supplementary Figure**

**Fig. S1.** Flowchart. HAP: hospital-acquired pneumonia, EOL: end-of-life, DNR: do not resuscitate.


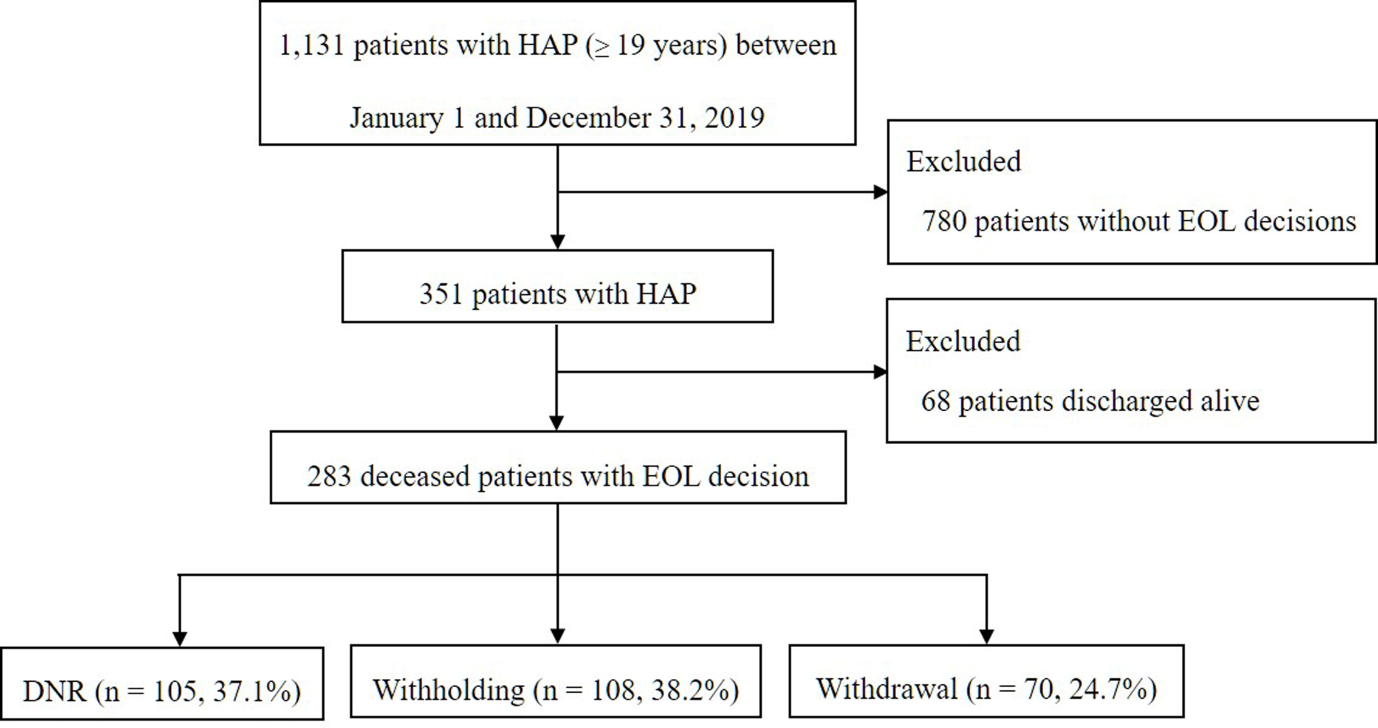


**Fig. S2.** Kaplan-Meier curve analysis for 60-day survival according to malignancy (a) Patients with malignancy. (b) Patients without malignancy.


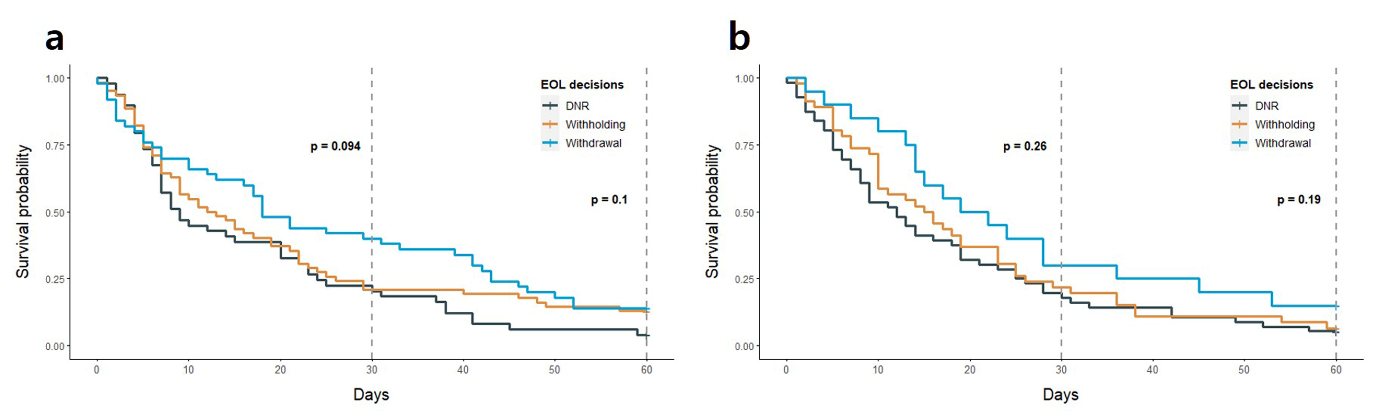

Supplement: Supplementary file 1 — Additional file 1: Fig. S1. Flowchart. HAP: hospital-acquired pneumonia, EOL: end-of-life, DNR: do not resuscitate. Fig. S2. Kaplan-Meier curve analysis for 60-day survival according to malignancy (a) Patients with malignancy. (b) Patients without malignancy. [file 12910_2023_931_MOESM1_ESM.docx]
