# Supplementary material for: Comparison of the end-of-life decisions of patients with hospital-acquired pneumonia after the enforcement of the life-sustaining treatment decision act in Korea
Source: BMC Med Ethics. 2023 Jul 18;24:52. doi: 10.1186/s12910-023-00931-y (PMC10353089; doi:10.1186/s12910-023-00931-y)
Supplement: Supplementary file 2 — Additional file 2: Supplementary Table 1. Pairwise comparison of the overall cohort. Supplementary Table 2. Pairwise comparison of ICU patients. [file 12910_2023_931_MOESM2_ESM.docx]

Supplementary Table 1. Pairwise comparison of the overall cohort

| Variables | DNR vs. Withholding | DNR vs. Withdrawal | Withholding vs. Withdrawal |
| --- | --- | --- | --- |
| Age (years) | 1.000 | 0.500 | 0.210 |
| Male, n (%) | 1.000 | 1.000 | 1.000 |
| Body mass index (kg/m^2^) | 1.000 | 1.000 | 1.000 |
| Comorbidities, n (%) |  |  |  |
| Diabetes | 0.366 | 1.000 | 1.000 |
| Cardiovascular disease | 0.175 | 1.000 | 1.000 |
| Chronic lung disease | 0.279 | 1.000 | 0.118 |
| Chronic neurological disease | 0.393 | 1.000 | 0.342 |
| Chronic kidney disease | 1.000 | 0.345 | 1.000 |
| Chronic liver disease | 1.000 | 0.241 | 0.125 |
| Hematological malignancy | 1.000 | 1.000 | 0.594 |
| Solid malignant tumors | 0.158 | 0.019 | 1.000 |
| Connective tissue disease | 1.000 | 1.000 | 1.000 |
| Immunocompromized | 1.000 | 0.696 | 0.555 |
| Reasons for admission, n (%) | 1.000 | 0.053 | 0.297 |
| Diagnostic work-up |  |  |  |
| Medical disease treatment |  |  |  |
| Elective operation |  |  |  |
| Emergency operation |  |  |  |
| Charlson Comorbidity Index | 0.031 | 0.131 | 1.000 |
| Clinical Frailty Scale | 0.920 | 1.000 | 1.000 |
| SOFA score | 0.790 | 1.000 | 1.000 |
| Location of diagnosis, n (%) | 0.666 | 1.000 | 1.000 |
| General ward |  |  |  |
| ICU |  |  |  |
| Artificial airway, n (%) | 0.363 | 0.651 | 1.000 |
| Tube feeding, n (%) | 1.000 | 1.000 | 1.000 |
| Impaired consciousness, n (%) | 0.354 | 0.048 | 1.000 |
| Impaired cough, n (%) | 1.000 | 0.008 | 0.008 |
| Sepsis, n (%) | 1.000 | 0.306 | 0.537 |
| Ventilator-associated pneumonia, n (%) | 0.296 | 0.936 | 1.000 |
| MDR pathogens, n (%) | 0.191 | 0.008 | 0.585 |
| ICU admission, n (%) | 0.942 | 0.056 | 0.510 |
| Hospital length of stay, days | 0.620 | 0.440 | 1.000 |
| Onset of HAP to death, days | 0.747 | 0.034 | 0.309 |
| 30-day mortality, n (%) | 1.000 | 0.038 | 0.097 |
| 60-day mortality, n (%) | 0.642 | 0.161 | 1.000 |

EOL: end-of-life; DNR: do not resuscitate; SOFA: Sequential Organ Dysfunction Assessment; MDR: multidrug-resistant; ICU: intensive care unit; HAP: hospital-acquired pneumonia.

Supplementary Table 2. Pairwise comparison of ICU patients

| Variables | DNR vs. Withholding | DNR vs. Withdrawal | Withholding vs. Withdrawal |
| --- | --- | --- | --- |
| Reasons for ICU admission, n (%) | 0.771 | 1.000 | 1.000 |
| Respiratory failure |  |  |  |
| Septic shock |  |  |  |
| Others |  |  |  |
| SOFA score |  |  |  |
| Within 24 h | 1.000 | 1.000 | 1.000 |
| At 48 h | 0.89 | 1.000 | 1.000 |
| Therapy within 24 h, n (%) |  |  |  |
| Vasopressors | 0.417 | 0.954 | 1.000 |
| Inotropes | 1.000 | 0.900 | 0.275 |
| HFNC | 1.000 | 1.000 | 1.000 |
| MV | 1.000 | 1.000 | 1.000 |
| Therapy during ICU stay, n (%) |  |  |  |
| HFNC | 1.000 | 1.000 | 1.000 |
| MV | 1.000 | 1.000 | 1.000 |
| RRT | 0.018 | 1.000 | 1.000 |
| ECMO | 1.000 | 0.540 | 0.275 |
| Events during ICU stay, n (%) |  |  |  |
| Ventilator-associated pneumonia | 1.000 | 1.000 | 1.000 |
| Catheter-related infection | 0.354 | 0.528 | 1.000 |
| Urinary tract infection | 1.000 | 1.000 | 1.000 |
| ARDS | 0.636 | 1.000 | 1.000 |
| Arrhythmia | 0.204 | 0.582 | 1.000 |
| Bleeding requiring intervention | 0.837 | 0.528 | 1.000 |
| Cardiopulmonary resuscitation | 1.000 | 1.000 | 1.000 |
| ICU duration, days | 1.000 | 0.71 | 0.58 |
| MV duration, days | 1.000 | 1.000 | 1.000 |
| ICU mortality | 0.157 | 0.168 | 1.000 |
| 30-day mortality | 1.000 | 0.054 | 0.062 |
| 60-day mortality | 1.000 | 1.000 | 1.000 |

ICU: intensive care unit; DNR: do not resuscitate; SOFA: Sequential Organ Dysfunction Assessment; HFNC: high-flow nasal cannula; MV: mechanical ventilation; RRT: renal replacement therapy; ECMO: extracorporeal membrane oxygenation; ARDS: acute respiratory distress syndrome.
